# Metabolic dysfunction and obesity‐related cancer: Results from the cross‐sectional National Health and Nutrition Examination Survey

**DOI:** 10.1002/cam4.4912

**Published:** 2022-06-19

**Authors:** Maci Winn, Prasoona Karra, Benjamin Haaland, Jennifer A. Doherty, Scott A. Summers, Michelle L. Litchman, Marc J. Gunter, Mary C. Playdon, Sheetal Hardikar

**Affiliations:** ^1^ Department of Population Health Sciences University of Utah Salt Lake City Utah USA; ^2^ Huntsman Cancer Institute University of Utah Salt Lake City Utah USA; ^3^ Department of Nutrition and Integrative Physiology University of Utah Salt Lake City Utah USA; ^4^ University of Utah College of Nursing Salt Lake City Utah USA; ^5^ Nutrition and Metabolism Branch International Agency for Research on Cancer Lyon France; ^6^ Fred Hutchinson Cancer Research Center Seattle Washington USA

**Keywords:** cancer, metabolic dysfunction, metabolic syndrome, obesity

## Abstract

**Background:**

Metabolic syndrome (MetS), a group of risk factors that define metabolic dysfunction in adults, is strongly associated with obesity and is an emerging risk factor for cancer. However, the association of MetS and degree of metabolic dysfunction with obesity‐related cancer is unknown.

**Methods:**

Using National Health and Nutrition Examination Survey data from 1999 to 2018, we identified 528 obesity‐related cancer cases and 18,972 cancer‐free participants. MetS was defined as the presence of or treatment for ≥3 of hyperglycemia, hypertension, hypertriglyceridemia, low HDL–cholesterol, and abdominal obesity. A metabolic syndrome score (MSS) was computed as the total number of abnormal MetS parameters to determine the severity of metabolic dysfunction. Odds ratios (ORs) and 95% confidence intervals (CIs) were estimated using multivariable logistic regression models, adjusting for sociodemographic and lifestyle factors.

**Results:**

About 45.7% of obesity‐related cancer cases were classified as having MetS compared with only 33.0% of cancer‐free participants. Overall, MetS and MSS were not associated with obesity‐related cancer. However, MSS was associated with higher obesity‐related cancer risk among participants under 50 years of age (OR [95% CI] = 1.28 [1.08–1.52]). When evaluating MSS categorically, compared with healthy participants with no abnormal MetS parameters (MSS = 0), participants with one or two abnormal parameters had a statistically significant higher risk of obesity‐related cancer (OR [95% CI] = 1.73 [1.06–2.83]).

**Conclusions:**

Metabolic dysfunction is associated with a higher risk of obesity‐related cancer, particularly in young adults under 50 years of age, and among participants with one or two abnormal metabolic parameters. A more accurate indicator of metabolic dysfunction, beyond metabolic syndrome, is needed to better assist in stratifying individuals for obesity‐related cancer risk.

## Graphical abstract

Participants with mild metabolic dysfunction i.e., 1‐2 abnormal metabolic syndrome (MetS) parameters, especially participants younger than 50 years, have a higher risk of obesity‐related cancer (ORC) compared to participants with zero abnormal MetS parameters. Traditionally, these individuals would not have been clinically diagnosed with MetS or perceived to be at higher risk for ORC, suggesting that measures beyond the clinical MetS diagnosis need to be considered to stratify individuals with respect to their ORC risk.

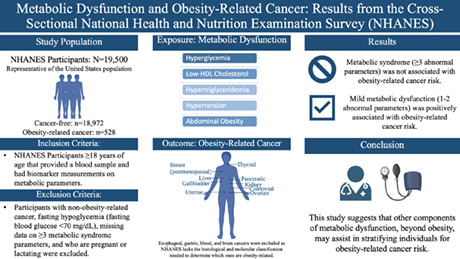

## 1 INTRODUCTION

There is overwhelming evidence that obesity increases the risk of several cancers, including breast, colorectal, uterine, ovarian, thyroid, kidney, pancreatic, liver, gallbladder, multiple myeloma, meningioma, gastric (cardia), and esophageal (adenocarcinoma) cancer.^1^ In the United States (U.S.), although the overall cancer incidence has been decreasing since the 1990s, obesity‐related cancer, which makes up 40% of all cancers, has been increasing.^2^ Several mechanisms may drive the etiology of obesity‐related cancer, including but not limited to inflammation,^3^ changes in metabolism,^4^ hormone signaling,^3^ microbiome composition,^5^ and insulin resistance.^6^ Indeed, an increasing number of studies suggest that poor overall metabolic health may contribute to obesity‐related cancer risk.^1^, ^7^, ^8^

Metabolic syndrome (MetS), a group of risk factors comprised of any three of hyperglycemia, hypertriglyceridemia, low high‐density lipoprotein‐cholesterol (HDL‐C), hypertension, and abdominal obesity, is currently used as a clinical indicator of metabolic health in adults.^9^ MetS, frequently used to estimate the risk of cardiovascular disease^10^ and diabetes,^11^ has more recently been studied as a risk factor for cancer. MetS has demonstrated an association with obesity‐related cancer in previous studies, including a recent large meta‐analysis with 38,940 cancer cases.^12^, ^13^, ^14^, ^15^, ^16^, ^17^, ^18^, ^19^ However, the significance and strength of these associations may vary by study population, sex, and cancer site. Metabolic dysfunction parameters, including hyperglycemia, hypertension, insulin resistance, and obesity, have demonstrated an independent association with obesity‐related cancer.^7^, ^16^, ^19^, ^20^, ^21^ However, less is known about their additive effect and incremental contribution to cancer risk. The purpose of this study was to determine the prevalence of MetS and metabolic dysfunction, indicated by one or two abnormal MetS parameters (thus not amounting to clinical diagnosis of MetS), in a large US‐representative population and to determine their association with obesity‐related cancer.

## 2 MATERIALS AND METHODS

### 2.1 Study population

We used data from the National Health and Nutrition Examination Survey,^22^ an ongoing population‐based cross‐sectional study conducted by the Center for Disease Control and Prevention (CDC) in the U.S. After written informed consent, data on participants’ sociodemographic characteristics (through questionnaires), anthropometric measurements (through physical exams), and biomarker values (from biospecimens) are collected. Study protocols are approved by the Institutional Review Boards at the CDC and these data are made publicly available. For the current analysis, we downloaded relevant NHANES data sets for years 1999–2018.

### 2.2 Inclusion and exclusion criteria

All participants in the NHANES database 18 years of age or older that provided a fasting blood sample were eligible to be included in the current analysis. As the MetS definition is based on the presence of three or more abnormal parameters, we excluded participants with three or more missing values for the MetS criteria (*N* = 2,226 [10.3%]). We also excluded participants with a cancer diagnosis other than obesity‐related cancer (*N* = 3,325 [3.4%]), fasting hypoglycemia (fasting blood glucose <70 mg/dl, *N* = 104 [0.11%]), and pregnant or lactating women (*N* = 1,857 [1.9%]). Additionally, despite being obesity related, we had to exclude all esophageal, gastric, blood, and brain cancers as NHANES lacks the histological and molecular classification needed to determine which ones are obesity related (*N* = 107 [0.11%]). After all exclusions, the analytical data set included data on 19,500 study participants.

### 2.3 Exposure classification

MetS was defined using the National Cholesterol Education Program’s Adult Treatment Panel III (NCEP ATPIII) criteria^9^ as the presence of three or more of the following: drug treatment of hyperglycemia or fasting blood glucose (FBG) ≥100 mg/dl, drug treatment for hypertension, diastolic blood pressure (DBP) ≥85 mmHg, or systolic blood pressure (SBP) ≥130 mmHg, abdominal obesity (waist circumference [WC] >88 cm [female] or >102 cm [male]), drug treatment for hypertriglyceridemia or triglycerides ≥150 mg/dl, or drug treatment of low HDL‐C, HDL‐C <50 mg/dl (female), or HDL‐C <40 mg/dl (male) (Table 1).

**TABLE 1:** Metabolic syndrome diagnosis and metabolic syndrome score (MSS) criteria

Measurement	Metabolic syndrome^a^/MSS^b^
Fasting Blood Glucose	>100 mg/dl or on drug treatment for hyperglycemia
HDL‐cholesterol	<40 mg/dl (male), <50 mg/dl (female) or on drug treatment for low‐HDL
Triglycerides	≥150 mg/dl or on drug treatment for hypertriglyceridemia
Blood pressure	DBP >85 mmHg or SBP >130 mmHg or on drug treatment for hypertension
Waist Circumference	≥102 cm (male), ≥88 cm (female)
	**Other metabolic dysfunction measures**
HOMA‐IR	[FBG (mg/dl) * insulin (mU/ml)/405]^c^
HbA1c	Normal: <5.7%, Prediabetic: 5.7%‐6.4%, Diabetic ≥6.5%^d^

Abbreviations: BMI, body mass index; DBP, diastolic blood pressure; FBG, fasting blood glucose; HbA1c, hemoglobin A1c; HDL‐cholesterol, high‐density lipoprotein‐cholesterol; HOMA‐IR, homeostatic model assessment of insulin resistance; SBP, systolic blood pressure.

^a^ Metabolic syndrome: ≥3 abnormal parameters; Defined using NCEP ATP III 2005 criteria.

^b^ Metabolic Syndrome Score (MSS): one point for each abnormal metabolic syndrome parameter.

^c^ Matthews et al., 1985.

^d^ American Diabetes Association.

Prevalent MetS was identified using questionnaire information, anthropometric data, and laboratory values. Hyperglycemia was determined by FBG concentration, drug treatment of hyperglycemia, or self‐report of a prior history of diabetes. The presence of hypertension was calculated by an average of at least two SBP or DBP readings, drug treatment for hypertension, or self‐report of a prior history of hypertension. In concordance with previous studies, participants with only one available blood pressure reading were not labeled as having hypertension due to the potential for inaccuracy.^23^, ^24^ The presence of hypertriglyceridemia and low HDL‐C were determined using drug treatment of dyslipidemia or laboratory values for triglycerides and HDL‐C. Abdominal obesity was defined using WC. Insulin resistance and HbA1c were evaluated as additional measures of metabolic dysfunction. Insulin resistance was measured using the Homeostatic Model Assessment for Insulin Resistance (HOMA‐IR) (FBG [mg/dl]*insulin [mU/ml]/405).^25^ High‐sensitivity C‐reactive protein (hsCRP) was considered as another potential marker of metabolic dysfunction, however, this biomarker was only available in a subset of NHANES participants for the years 2015–2018 (only 12.0% of our study participants), therefore, we did not evaluate hsCRP as an independent indicator of metabolic dysfunction.

The metabolic syndrome score (MSS), calculated as the total number of abnormal MetS components, was computed to determine if the number of abnormal MetS criteria present was associated with obesity‐related cancer risk, beyond the presence of MetS itself. This provides a score rather than a binary measure of metabolic dysfunction, allowing us to evaluate incremental associations of metabolic dysfunction with obesity‐related cancer. The MSS ranged from a minimum possible value of 0 (no abnormal MetS parameters) to a maximum of 5 (all MetS parameters abnormal).

### 2.4 Outcome classification

Participants were classified as having obesity‐related cancer using the NHANES medical condition questionnaires. Obesity‐related cancer sites were determined as defined by the National Cancer Institute (NCI)^1^ and included breast, colorectal, uterine, ovarian, thyroid, kidney, liver, pancreatic, gallbladder, multiple myeloma, meningioma, esophageal adenocarcinoma, and gastric cardia cancer.^1^ However, all types of blood, brain, esophageal, and stomach cancers in NHANES are combined into one group without histologic or molecular subtype classification, thus, as outlined in the methods above, we excluded these cancers from our analysis. The following two questions were used to define the presence of obesity‐related cancer: (1) “Have you ever been told by a doctor or other health professional that you had cancer or a malignancy of any kind?” and (2) “What kind of cancer?”

### 2.5 Covariates

Analyses were adjusted for age (<50, 50–59, 60–69, 70–79, and ≥80), sex, race/ethnicity (non‐Hispanic White, non‐Hispanic Black, Mexican American/Other Hispanic, and Other [including non‐Hispanic Asian and all non‐Hispanic participants who reported races other than Black, Asian, or White]), education level (grade school, high school, and college), annual household income (<$35,000, $35,000–$74,999, ≥$75,000, and not reported), smoking status (never, former, and current), alcohol use (yes/no), sedentary behavior (daily hours available 2007–2018; daily computer, video game, and TV hours used for 1999–2006), weekly physical activity level (low/no, moderate, and vigorous activity), daily calorie intake, and survey year. As WC was statistically significantly correlated with BMI (*r* ^2^ = 0.90, *p* < 0.0001), we did not adjust for BMI in the current analysis.

### 2.6 Statistical analysis

Descriptive statistics and correlations between all MetS components were computed. Odds ratios (ORs) and 95% confidence intervals (95% CIs) for obesity‐related cancer risk and association with the various parameters of metabolic health (each component of MetS, HOMA‐IR, and HbA1c), the presence of MetS, and severity of MetS were estimated using multivariable logistic regression models adjusting for (1) age and sex and (2) age, sex, race/ethnicity, education level, annual household income, smoking status, alcohol use, daily hours sedentary, weekly physical activity, daily calorie intake, and survey year. To determine the degree to which the associations between MetS, MSS, and obesity‐related cancer were modified by age, sex, race/ethnicity, and BMI, subgroup analyses were conducted using multivariable‐adjusted logistic regression models. Because of the complex survey design of NHANES, all estimates were weighted according to the NHANES analytic guidelines to be representative of the U.S. civilian noninstitutionalized‐resident population.^26^ As our main exposure, MetS includes multiple biomarker measurements, we used “wtsaf4yr” and “wtsaf2yr” weights, as indicated to be used when including values obtained from fasting blood samples. Implausible laboratory measurements (BMI >130 kg/m^2^, DBP < 40 mmHg) were changed to missing. Missing covariate values were analyzed as a separate category. In sensitivity analyses, we evaluated the risk of obesity‐related cancer with MetS in a complete case analysis, excluding all participants with missing MetS parameter data. All statistical analyses were carried out using SAS Studio, and significance was determined at α = 0.05.

## 3 RESULTS

In the current analyses, we evaluated associations of MetS and other metabolic dysfunction parameters with obesity‐related cancer among NHANES participants from 1999 to 2018. Baseline characteristics with weighted proportions are summarized in Table 2. Overall, there were 19,500 participants, 528 (2.5%) of whom reported a history of obesity‐related cancer, whereas 18,972 were cancer‐free participants. Compared with cancer‐free participants, participants with obesity‐related cancer were older (16.5% vs. 3.0%, ≥80 years), female (90.4% vs. 50.0%), and non‐Hispanic White (78.9% vs. 67.1%). Female obesity‐related cancer cases were more likely to be postmenopausal (87.0% vs. 45.1%) and more likely to have used hormone replacement therapy (HRT; 39.7% vs. 20.5%). No differences were observed between groups by any other characteristics.

**TABLE 2:** Demographic information for the National Health and Nutrition Examination Survey participants with a prior history of obesity‐related cancer (ORC) compared with cancer‐free participants (*N* = 19,500)

	No cancer, *n* = 18,972		ORC cases, *n* = 528	
	*Actual frequency/mean*	*%/SE*	*Actual frequency/mean*	*%/SE*
Age in years^a^				
<50	10,772	62.4	72	19.3
50–59	2,785	17.3	70	14.4
60–69	2,819	11.5	136	25.3
70–79	1,643	5.9	143	24.5
≥80	953	3.0	107	16.5
Sex				
Female	9,418	50.0	458	90.4
Male	9,554	50.0	70	9.6
Race/ethnicity				
White (non‐Hispanic)	8,013	67.1	313	78.9
Black (non‐Hispanic)	3,954	11.8	83	8.7
Mexican American/Other Hispanic	5,402	14.3	108	8.5
Other^b^	1,603	6.8	24	3.8
Income				
<$35,000	8,955	37.2	267	43.0
$35,000–$75,000	5,498	32.1	153	32.5
>$75,000	2,479	18.0	59	13.1
Education				
<High School	5,538	19.3	155	20.8
High School	4,488	24.1	125	27.7
>High School	8,922	56.5	248	51.6
Smoking				
Never	9,701	52.2	284	51.6
Former	4,219	23.2	180	34.7
Current	3,769	21.5	63	13.6
Body mass index (kg/m^2^)				
<25 (normal weight)	6,037	32.7	153	33.7
25–<30 (overweight)	6,221	32.6	160	28.9
≥30 (obese)	6,442	33.5	205	35.2
Total daily calorie intake	2,084	10	1,649	30
Physical activity				
No/low activity	6,567	29.4	243	38.8
Moderate/vigorous activity	12,123	69.4	263	57.6
Sedentary hours				
<5 h	10,914	56.6	284	53.6
≥5 h	8,052	43.4	244	46.4
Alcohol use				
No	4,317	19.7	191	33.4
Yes	10,467	61.3	254	50.6
Menopausal status^c^				
Premenopausal	4,110	47.3	33	8.3
Postmenopausal	4,509	45.1	397	87.0
HRT use^c^				
No	6,190	68.3	270	54.9
Yes	1,746	20.5	157	39.7
Years Since Cancer Diagnosis	N/A	N/A	12.0	0.53

Notes

Data was missing for the following: Income: 10.7%, Education: 0.1%, Smoking: 6.6%, BMI: 1.4%, Physical Activity: 1.6%, Sedentary Behavior: 0.0%, Alcohol: 21.9%, Menopausal Status: 8.4%, HRT: 15.3%, Years Since Cancer Diagnosis: 0.2%.

Abbreviations: HRT, hormone replacement therapy; SD, standard error.

^a^ Participants with age ≥ 85 are recorded as age = 85 in the NHANES datasets.

^b^ The “other” race category in NHANES includes non‐Hispanic Asian and all non‐Hispanic persons that reported races other than Black, Asian, or White.

^c^ Among female study participants only.

Table 3 outlines the differences in metabolic dysfunction parameters between obesity‐related cancer cases and cancer‐free participants. About half (45.7%) of obesity‐related cancer cases had MetS, compared with 33.0% of cancer‐free participants. A larger proportion of obesity‐related cancer cases had abnormal levels for each MetS criteria except for low HDL‐C. Similarly, obesity‐related cancer cases had a higher average HOMA‐IR and HbA1c compared with cancer‐free participants. The difference was particularly marked for abdominal obesity (68.0% vs. 51.8%), hypertension (53.6% vs. 32.9%), and hyperglycemia (57.9% vs. 43.4%). Adjusted ORs and 95% CIs for obesity‐related cancer risk were also estimated for each metabolic dysfunction parameter classified as a binary variable based on the clinical definition of MetS using the NCEP ATPIII criteria (Table 3). In fully adjusted models, hyperglycemia, hypertriglyceridemia, hypertension, HOMA‐IR, and HbA1c were associated with a higher risk of obesity‐related cancer, however, none were statistically significant. We ran additional models using clinical cutoff values to create categorical HOMA‐IR and HbA1c variables. Currently, there is no clear consensus on HOMA‐IR cutoff values. The original HOMA‐IR model by Matthews et al., in 1985 defined 2.5 as a general cutoff.^25^ However, Lam et al. observed that the cutoff of 4.0 had the lowest misclassification rate for insulin resistance.^27^ We used both cutoff recommendations to categorize participants into low‐risk, moderate‐risk, and high‐risk groups. Using this categorical variable, we observed a similar relationship with obesity‐related cancer risk as the continuous variable, with a slightly stronger relationship for the moderate‐risk group (OR 1.16, 95% CI 0.88–1.53), however, none of these associations reached statistical significance. Moreover, further research in more diverse populations is still needed to determine valid cutoff values for HOMA‐IR. HbA1c cutoff values were determined by the National Institute of Diabetes and Digestive and Kidney Diseases (NIDDK) as normal (<5.7), prediabetic (5.7–6.5), and diabetic (>6.5). In this analysis, we observed a statistically significant higher risk of obesity‐related cancer in participants with a prediabetic HbA1c value compared to participants with a normal HbA1c (OR 1.38, 95% CI 1.07–1.79). The association with diabetic values of HbA1c was less strong and did not reach statistical significance. Thus, moderate levels of glycated hemoglobin may be an important factor in determining obesity‐related cancer risk. These results are provided in Table S1. In subgroup analyses by cancer site (Figure S1), HOMA‐IR was statistically significantly associated with colorectal cancer risk (OR [95% CI]: 1.02 [1.01–1.03]). We did not observe any associations between metabolic parameters and other cancer types.

**TABLE 3:** Means, standard deviations, odd ratios (OR), and 95% confidence intervals (CI) of metabolic dysfunction parameters and anthropometric measurements for the National Health and Nutrition Examination Survey participants with a prior history of obesity‐related cancer (ORC) diagnosis compared to cancer‐free participants (*N* = 19,500)

Metabolic parameters^a^	Cancer‐free participants, *n* = 18,972	ORC cases, *n* = 528				
		*Age and sex adjusted*			*Fully adjusted* ^d^	
	*N/mean(%/SE)*	*N/mean(%/SE)*	OR^b^	95% CI^b^	OR	95% CI
Hyperglycemia	8,851 (43.4)	328 (57.9)	1.24	1.01–1.51	1.25	1.03–1.52
Low HDL‐C^b^	5,673 (33.0)	168 (32.1)	0.90	0.72–1.13	0.89	0.71–1.12
Hypertriglyceridemia	5,565 (29.8)	202 (40.0)	1.25	0.97–1.61	1.23	0.95–1.60
Hypertension	7,057 (32.9)	311 (53.6)	1.09	0.86–1.39	1.10	0.86–1.40
Abdominal Obesity	9,550 (51.8)	356 (68.0)	0.93	0.71–1.21	0.91	0.70–1.17
HOMA‐IR^c^	3.35 (0.05)	3.69 (0.23)	1.01	0.99–1.02	1.01	1.00–1.02
HbA1c	5.53 (0.01)	5.80 (0.04)	1.07	0.98–1.16	1.10	1.01–1.20

Notes

Data was missing for low HDL‐cholesterol (1990 cancer‐free, 57 cases), hypertriglyceridemia (193 cancer‐free, 10 cases), hypertension (10 cancer‐free), abdominal obesity (593 cancer‐free, 32 cases), and HOMA‐IR (452 cancer‐free, 20 cases).

^a^ Defined using NCEP ATP III 2005 criteria.

^b^ Abbreviations: HbA1c, glycated hemoglobin; HDL‐C, HDL‐Cholesterol; HOMA‐IR, homeostatic model assessment of insulin resistance; SE, standard error.

^c^ HOMA‐IR = fasting blood glucose (mg/dl) × fasting insulin (mU/ml)/405.

^d^ Model adjusted for age, sex, race/ethnicity, education level, annual household income, smoking status, alcohol use, daily hours sedentary, weekly physical activity level, daily calorie intake, and survey year.

Mutual correlations between the various metabolic dysfunction parameters are outlined in Table 4. Most parameters were weakly correlated with each other (range −0.35 to +0.43). As expected, HDL‐C was inversely correlated with the other parameters, and FBG was highly correlated with HbA1C (*r* = 0.84).

**TABLE 4:** Correlation coefficients of metabolic dysfunction parameters in the National Health and Nutrition Examination Survey participants (*N* = 19,500)^a^

	FBG^a^	HDL‐C^a^	TG^a^	SBP^a^	DBP^a^	WC^a^	HOMA‐IR^a^	HbA1c^a^
FBG^a^	1.00							
HDL‐C^a^	−0.14	1.00						
TG^a^	0.24	−0.35	1.00					
SBP^a^	0.19	0.01	0.11	1.00				
DBP^a^	0.03	−0.06	0.10	0.30	1.00			
WC^a^	0.25	−0.32	0.20	0.23	0.14	1.00		
HOMA‐IR^a^	0.43	−0.19	0.20	0.09	0.03	0.33	1.00	
HbA1c^a^	0.84	−0.11	0.21	0.20	0.02	0.27	0.35	1.00

Abbreviations: FBG, Fasting blood glucose (plasma; HbA1c, glycated hemoglobin; HDL‐C, HDL‐Cholesterol (mg/dl); HOMA‐IR, homeostatic model assessment of insulin resistance; mg/dl); SBP/DBP, Systolic and Diastolic Blood Pressure (mmHG); TG, Triglycerides (mg/dl); WC, Waist Circumference (cm).

^a^ The *p*‐value was <.001 for all parameter correlations, except between HDL‐C and SBP (*p* = 0.11).

**FIGURE 1:**
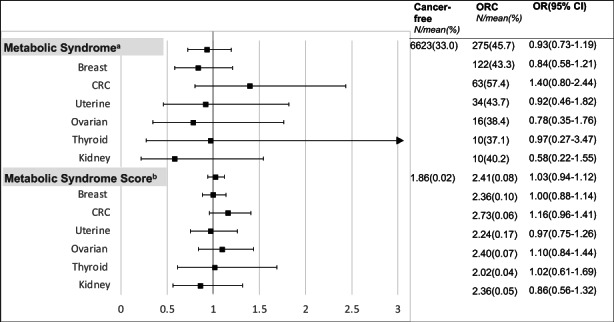
Adjusted odds ratios and 95% confidence intervals for the risk of obesity‐related cancer with metabolic syndrome, and metabolic syndrome score compared to participants with no cancer, overall and by cancer site in the National Health and Nutrition Examination Survey (NHANES) participants 1999–2018 (*N* = 19,500)^c*d*^. ^a^Metabolic syndrome: ≥3 abnormal parameters; Defined using NCEP ATP III 2005 criteria. ^b^Metabolic Syndrome Score: one point for each abnormal metabolic syndrome parameter. ^c^All analyses were adjusted for age, sex, race/ethnicity, education level, annual household income, smoking status, alcohol use, daily hours sedentary, weekly physical activity level, daily calorie intake, and survey year. ^d^Cancer sites: Breast (*N* = 258), colorectal (*N* = 116), uterine (*N* = 72), ovarian (*N* = 40), thyroid (*N* = 31), kidney (*N* = 30), liver (*N* = 4), pancreatic (*N* = 2), and gallbladder (*N* = 1).

Neither the presence of MetS nor the severity of MetS as indicated by increasing MSS was associated with obesity‐related cancer overall (MetS: OR 0.93, 95% CI 0.72–1.19, MSS: OR 1.03, 95% CI 0.94–1.12 per unit increase in MSS) (Table 5). A complete case analysis excluding participants with missing values for the MetS criteria showed similar results (Table S2). In subgroup analyses by cancer site, MetS and MSS were associated with a higher risk of colorectal cancer but this was not statistically significant (Figure 1). When evaluating MSS categorically, compared to healthy participants with no abnormal MetS parameters (MSS = 0), participants with one or two abnormal parameters had a 1.73 times statistically significant higher risk of obesity‐related cancer (OR 1.73 95% CI 1.06–2.83; Table 5). Having three or more parameters in the abnormal range, in line with the MetS definition, was also associated with a higher risk of obesity‐related cancer, although statistically nonsignificant, and the effect size was smaller (51% increase in odds [OR 1.51 95% CI 0.91–2.50] vs. 73% among those with one or two abnormal parameters). To determine which metabolic parameters were driving the higher risk of obesity‐related cancer, we evaluated different combinations of two MetS parameters compared to zero abnormal parameters. The combination of hypertriglyceridemia and hypertension was associated with a more than a two times higher risk of obesity‐related cancer, however, this was not statistically significant. Interestingly, the combination of low HDL‐cholesterol and central obesity was associated with a statistically significant 85% decreased risk of obesity‐related cancer (OR 0.15 95% CI 0.03–0.87; Table S3).

**TABLE 5:** Adjusted odds ratios (OR) and 95% confidence intervals (CI) for obesity‐related cancer (ORC) with metabolic syndrome and metabolic syndrome score in National Health and Nutrition Examination Survey participants (*N* = 19,500)

	Cancer‐free participants, *n* = 18,972 *N/mean(%/SE)* ^c^	ORC Cases, *n* = 528 *N/mean(%/SE)* ^c^	OR^d^	95% CI^d^	OR^e^	95% CI^e^
Metabolic Syndrome						
No	12349 (67.0)	253 (54.3)	REF	REF	REF	REF
Yes	6,623 (33.0)	275 (45.7)	0.94	0.73–1.20	0.93	0.72–1.19
Metabolic Syndrome Score (MSS)^a^	1.86 (0.02)	2.41 (0.08)	1.03	0.94–1.13	1.03	0.94–1.12
MSS categories^b^						
0	3,554 (20.7)	28 (7.1)	REF	REF	REF	REF
1 or 2	8,795 (46.3)	225 (47.2)	1.76	1.07–2.89	1.73	1.06–2.83
3, 4, or 5	6,623 (33.0)	275 (45.7)	1.53	0.91–2.58	1.51	0.91–2.50

^a^ MSS computed as a continuous variable, where each abnormal MetS parameter received a score of 1 and was summed to a total score out of 5.

^b^ MSS computed as a categorial variable, where the total score out of 5 (number of abnormal MetS parameters for each participant) was compared to healthy participants with no abnormal MetS parameters (ref = 0).

^c^ Abbreviation: SE, standard error.

^d^ Age and sex adjusted.

^e^ Model adjusted for age, sex, race/ethnicity, education level, annual household income, smoking status, alcohol use, daily hours sedentary, weekly physical activity level, daily calorie intake, and survey year.

**FIGURE 2:**
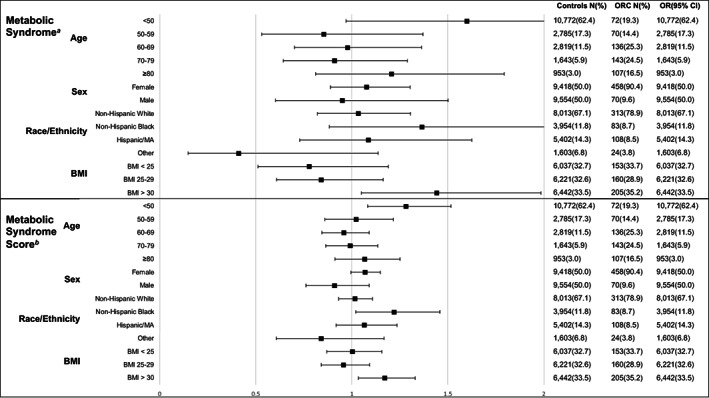
Adjusted odds ratios and 95% confidence intervals for the risk of obesity‐related cancer with metabolic syndrome and metabolic syndrome score stratified by age, sex, and race/ethnicity in the National Health and Nutrition Examination Survey participants 1999–2018 (*N* = 19,500)^c^. ^a^Metabolic syndrome: ≥3 abnormal parameters; Defined using NCEP ATP III 2005 criteria. ^b^Metabolic Syndrome Score: one point for each abnormal metabolic syndrome parameter. ^c^All analyses were adjusted for age, sex, race/ethnicity, education level, annual household income, smoking status, alcohol use, daily hours sedentary, weekly physical activity level, daily calorie intake, and survey year. MA, Mexican American.

Additional subgroup analyses were conducted to evaluate differences by age, sex, race/ethnicity, and BMI (Figure 2). There was a difference in the association with obesity‐related cancer by age such that among participants under 50 years of age, the presence of MetS, and a unit increase in MSS were associated with a 1.60 and 1.28 times higher risk of obesity‐related cancer, respectively, however, only the association with MSS was statistically significant (OR 1.28 95% CI 1.08–1.52). When stratified by race, a unit increase in MSS was statistically significantly associated with a 22% higher risk of obesity‐related cancer among non‐Hispanic Black participants (OR [95% CI]: 1.22 [1.02–1.46]). When stratified by BMI, MetS and MSS were both positively associated with obesity‐related cancer in obese participants (BMI ≥ 30) (OR [95% CI]: 1.44 [1.05–1.99], 1.17 [1.03–1.33], respectively). No differences were observed by sex.

We performed an additional analysis comparing normal weight and overweight cancer‐free participants to participants with obesity‐related cancer (Table S4). In general, cancer‐free participants that were overweight (body mass index ≥25 kg/m^2^) were older; 59.1% of cancer‐free overweight participants were under 50 years of age compared with 69.2% of normal weight cancer‐free participants. As expected, all metabolic parameter levels were worse for overweight cancer‐free participants compared with normal weight cancer‐free participants. Obesity‐related cancer participants were more likely to be older and female compared with overweight cancer‐free participants, owing to the association of age with cancer development and most obesity‐related cancers being cancers of the female reproductive system. Notably, metabolic parameters were very similar between obesity‐related cancer cases and overweight cancer‐free participants. Metabolic parameters were mostly worse for obesity‐related cancer cases, except, however, for HOMA‐IR and abdominal obesity, adding to the hypothesis that metabolic dysfunction may be an important component of cancer risk.

Because many obesity‐related cancers are reproductive cancers, sensitivity analyses were performed by restricting our data set to females and additionally adjusting for menopausal status and HRT use. We also performed a sensitivity analysis for overall obesity‐related cancer risk and breast cancer risk with MetS and MSS, restricting to postmenopausal breast cancer cases only (premenopausal breast cancers were excluded). No significant differences were observed in these analyses compared with the entire population (Tables S5 and S6). Also, as older individuals are more likely to both develop cancer and a metabolic dysfunction, we performed additional secondary analyses excluding all individuals under 30 and under 40 years of age in separate analyses; results were similar in magnitude and direction to our primary analyses (data not shown).

## 4 DISCUSSION

We examined the association between metabolic dysfunction and obesity‐related cancer in a nationally representative cohort comprised of 528 obesity‐related cancer cases and 18,972 cancer‐free participants. We observed a greater degree of metabolic dysfunction among those that reported obesity‐related cancer compared with cancer‐free participants. For example, almost half of obesity‐related cancer cases had MetS versus just over a third of cancer‐free individuals. Moreover, 93% of obesity‐related cancer cases had at least one abnormal MetS parameter compared with 79% of cancer‐free participants. Our results suggest that metabolic dysfunction even in a single MetS parameter, in contrast to the binary definition of MetS, was positively associated with the presence of obesity‐related cancer.

Proposed mechanisms to explain the relationship between MetS and obesity‐related cancer include alterations in hormone signaling, oxidative stress, and chronic low‐grade inflammation.^3^, ^4^ Obesity has been characterized by a broad inflammatory response that perpetuates insulin resistance ultimately resulting in elevated diabetes, cardiovascular disease, and cancer risk.^28^ Obesity‐associated inflammation results from mechanisms, including but not limited to excess adipose tissue‐stimulating cytokine release^3^ and excess lipopolysaccharide release from altered gut barrier function.^5^ Additionally, adipose‐induced insulin resistance and insulin‐like growth factor‐1 (IGF‐1) production can lead to hyperglycemia, excess circulating steroid hormones, and elevated free fatty acid levels, all of which contribute to metabolic dysfunction and tumor progression.^3^, ^6^ Here, we observed that only obesity (BMI≥30) was associated with obesity‐related cancer. This suggests that other components of metabolic dysfunction, such as dyslipidemia, hyperglycemia, and hypertension, while consequences of obesity are also independently associated with obesity‐related cancer.

Each of the MetS parameters is associated with cancer development.^12^, ^29^, ^30^, ^31^ Hyperglycemia and diabetes are associated with colorectal, breast, liver, pancreatic, uterine, and bladder cancer.^32^, ^33^, ^34^ Hyperglycemia results in insulin resistance and upregulation of IGF‐1. This may contribute to cancer development as well as tumor progression, as glucose is necessary for proliferating cells.^35^ Hypertension has been shown to increase the risk of renal,^30^ colorectal,^30^ meningioma,^36^ pancreatic,^37^ uterine,^38^ and esophageal^16^ cancers. The exact mechanisms behind these relationships are unknown, however, formation of reactive oxygen species and anti‐hypertensive treatment have been suggested as potential causes.^39^ We did not observe an association between hypertension and obesity‐related cancer. The difference in our results could potentially be due to changes in weight from cancer‐related mechanisms or treatment‐induced cardiotoxicity, causing alterations in hypertension status.^40^

Dyslipidemia is also an independent risk factor for obesity‐related cancer. *Radišauskas et al*. reported that although there are multiple studies linking total cholesterol levels to increased cancer risk,^30^ the strength and direction of this association are unclear. Total cholesterol is a combination of both low‐density and high‐density lipoproteins. Like the association of total cholesterol with cardiovascular risk, the opposing effect of low‐ and high‐density lipoproteins may neutralize the overall increased risk for cancer.^41^ Most studies evaluating the association of dyslipidemia and cancer have shown an increased risk with both high triglycerides and low HDL‐C.^42^, ^43^, ^44^, ^45^ Additionally, higher levels of HDL‐C may be a protective factor for obesity‐related cancer.^44^ The reasons behind this relationship are not entirely understood, but there is growing evidence that lipid metabolism is involved in tumor pathogenesis.^45^

Although the MetS parameters may have independent associations with obesity‐related cancer, it remains unclear whether these effects are additive and whether increasing severity of metabolic dysfunction increases cancer risk in a dose–response manner. Our study did not observe that the number of abnormal MetS parameters, calculated as MSS, was monotonically associated with obesity‐related cancer. However, compared with healthy participants (i.e., no abnormal MetS parameters), having one or two abnormal MetS criteria was statistically significantly associated with obesity‐related cancer. Traditionally, these individuals (~46%) would not have been clinically diagnosed with MetS. This suggests that mild metabolic dysfunction may increase cancer risk, and measures beyond the clinical MetS diagnosis need to be considered to stratify individuals with respect to their obesity‐related cancer risk.

Having MetS was not associated with obesity‐related cancer in this study. However, with evidence that MetS increases cancer‐related mortality,^31^ selection bias is a possibility if cancer survivors with MetS were less likely to participate in the NHANES study owing to poor health or increased cancer‐related mortality. Additionally, cancer cachexia could be prevalent in many cancer cases, resulting in a smaller waist circumference and fewer cases being categorized as having MetS. Although MSS is currently not a validated measure of metabolic dysfunction, there are some studies showing the potential additive or synergistic effects of the MetS parameters on cancer risk.^46^ A dose–response relationship between the number of MetS components and colorectal cancer risk has been reported.^32^ A similar metabolic risk score has previously demonstrated statistically significant associations with multiple obesity‐related cancers.^47^ Preclinical studies support these observations. For example, rats injected with a combination of glucose, lipids, and insulin have greater colorectal epithelial proliferation than those injected with insulin alone.^48^

We observed that metabolic dysfunction was associated with obesity‐related cancer among younger adults (<50 years). Recent reports show an increasing incidence of obesity‐related cancer in adolescents and young adults, specifically for colorectal, breast, kidney, uterine, pancreatic, and thyroid cancers.^49^, ^50^, ^51^ Improving metabolic health screening and interventions could be crucial for cancer prevention in the young adult population and warrants further investigation. We also observed associations between metabolic dysfunction and obesity‐related cancer in non‐Hispanic Black participants. However, further research in larger cohorts is needed, as the NHANES race/ethnicity data lacks the necessary specificity.

Our study has some limitations. Cancers were self‐reported and, therefore, there is a possibility of misclassification and recall bias. There may also be misclassification of the exposure resulting from imperfect biomarker measurement. However, we anticipate any such misclassification to be non‐differential and, therefore, will only bias the ORs towards the null. Another major limitation is the cross‐sectional nature of the data, thus precluding us from making inferences on temporality. Because of this, we are unable to preclude the temporality of obesity and cancer diagnoses in NHANES participants. Moreover, NHANES does not include data on cancer treatment. It is possible that cancer patients in this population experienced weight reduction after a cancer diagnosis, which could result in the absence of MetS and reverse causality in this study. Multiple mechanisms may be involved in such a weight loss, including reduced food intake, response to cancer treatment, and hypercatabolism resulting from inflammation, tumor metabolism, or insulin resistance.^52^, ^53^ Additionally, we could not include some obesity‐related cancers (multiple myeloma, meningioma, gastric cardia, esophageal adenocarcinoma) due to the lack of detailed histologic cancer classification within NHANES. However, these cancers are rare, and thus, their exclusion is unlikely to have significantly affected the results presented in this study. Last, this study is not powered to adequately examine associations by cancer site and by sex. However, we performed exploratory analyses by cancer site and sex to examine differences in both MetS and metabolic syndrome scores as well as by each metabolic dysfunction parameter. This study has several strengths. First, NHANES is a large and nationally representative sample with well‐validated questionnaires, medical examinations, and biomarker assessments. Second, MetS was defined based on a clinically accepted definition using detailed laboratory data and objective measures of adiposity. Third, we evaluated measures, such as MSS and HOMA‐IR, to further examine metabolic dysfunction beyond the strict clinical definition of MetS.

In conclusion, metabolic dysfunction is associated with higher obesity‐related cancer risk, particularly in young adults under 50 years of age and in participants with one or two abnormal metabolic parameters. Previous studies that have only considered those with MetS (≥3 abnormal parameters) as metabolically unhealthy may, in fact, be excluding many metabolically unhealthy individuals at a higher risk for obesity‐related cancer. Further research on metabolic dysfunction could provide insight into potential mechanisms between metabolic parameters and obesity‐related cancer and allow for better risk stratification and cancer prevention strategies in higher‐risk subgroups.

## AUTHORS’ CONTRIBUTION

Conceptualization: M.W., M.P., and S.H.; methodology: M.W., M.P., and S.H; formal analysis: M.W., M.P., S.H., and B.H.; writing—original draft preparation: M.W. and S.H.; writing—review and editing: M.W., P.K., J.D., S.S., B.H., M.L., M.G., M.P., and S.H; visualization: M.W., M.P., and S.H.; supervision: M.P. and S.H.

## CONFLICT OF INTEREST

S.A. Summers is a co‐founder and consultant with Centaurus Therapeutics. All other co‐authors do not declare any conflict of interest.

## ETHICS STATEMENT

NHANES is a publicly available data set approved by the National Center for Health Statistics institutional review board, and all participants provided written informed consent.

## Supporting information


Figure S1

Table S1‐S7

## Data Availability

Some or all data generated or analyzed during this study are included in this published article or in the data repositories listed in References. URL: https://wwwn.cdc.gov/nchs/nhanes/Default.aspx
