# Supplementary material for: Metabolic dysfunction and obesity‐related cancer: Results from the cross‐sectional National Health and Nutrition Examination Survey
Source: Cancer Med. 2022 Jun 19;12(1):606–18. doi: 10.1002/cam4.4912 (PMC9844618; doi:10.1002/cam4.4912)

## Slide 1
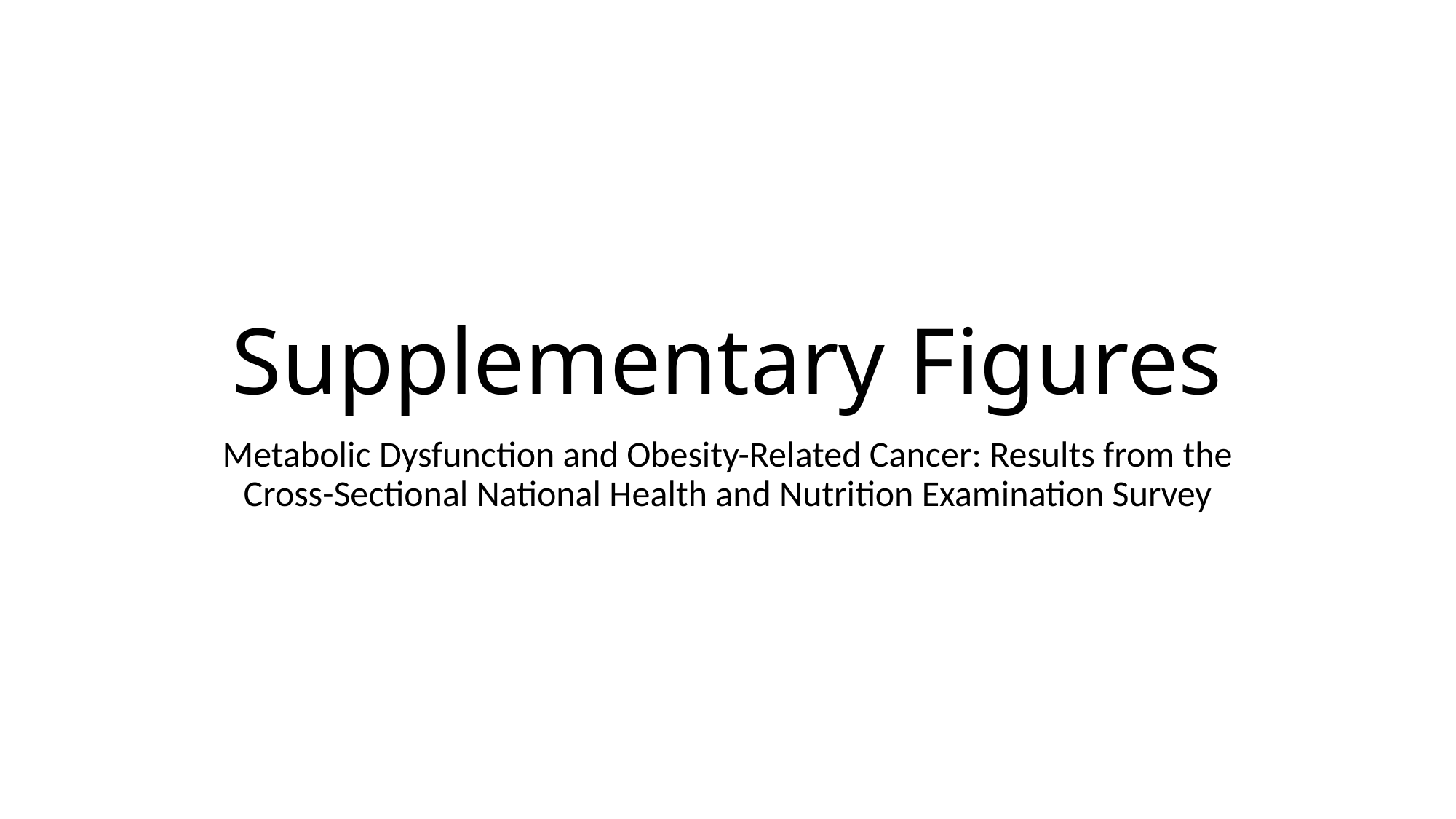

# Supplementary Figures
Metabolic Dysfunction and Obesity-Related Cancer: Results from the Cross-Sectional National Health and Nutrition Examination Survey

## Slide 2
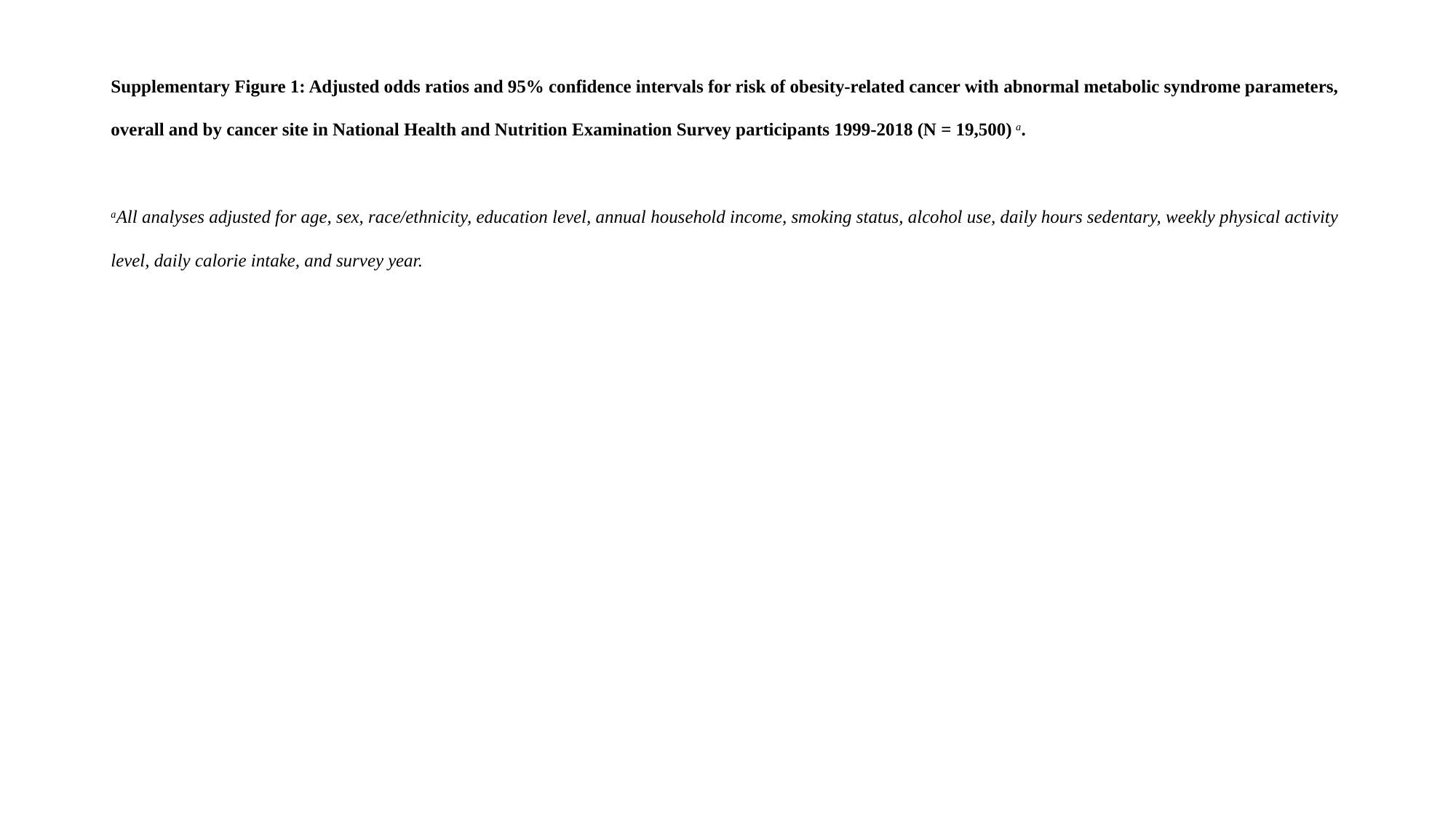

Supplementary Figure 1: Adjusted odds ratios and 95% confidence intervals for risk of obesity-related cancer with abnormal metabolic syndrome parameters, overall and by cancer site in National Health and Nutrition Examination Survey participants 1999-2018 (N = 19,500) a.
aAll analyses adjusted for age, sex, race/ethnicity, education level, annual household income, smoking status, alcohol use, daily hours sedentary, weekly physical activity level, daily calorie intake, and survey year.

## Slide 3
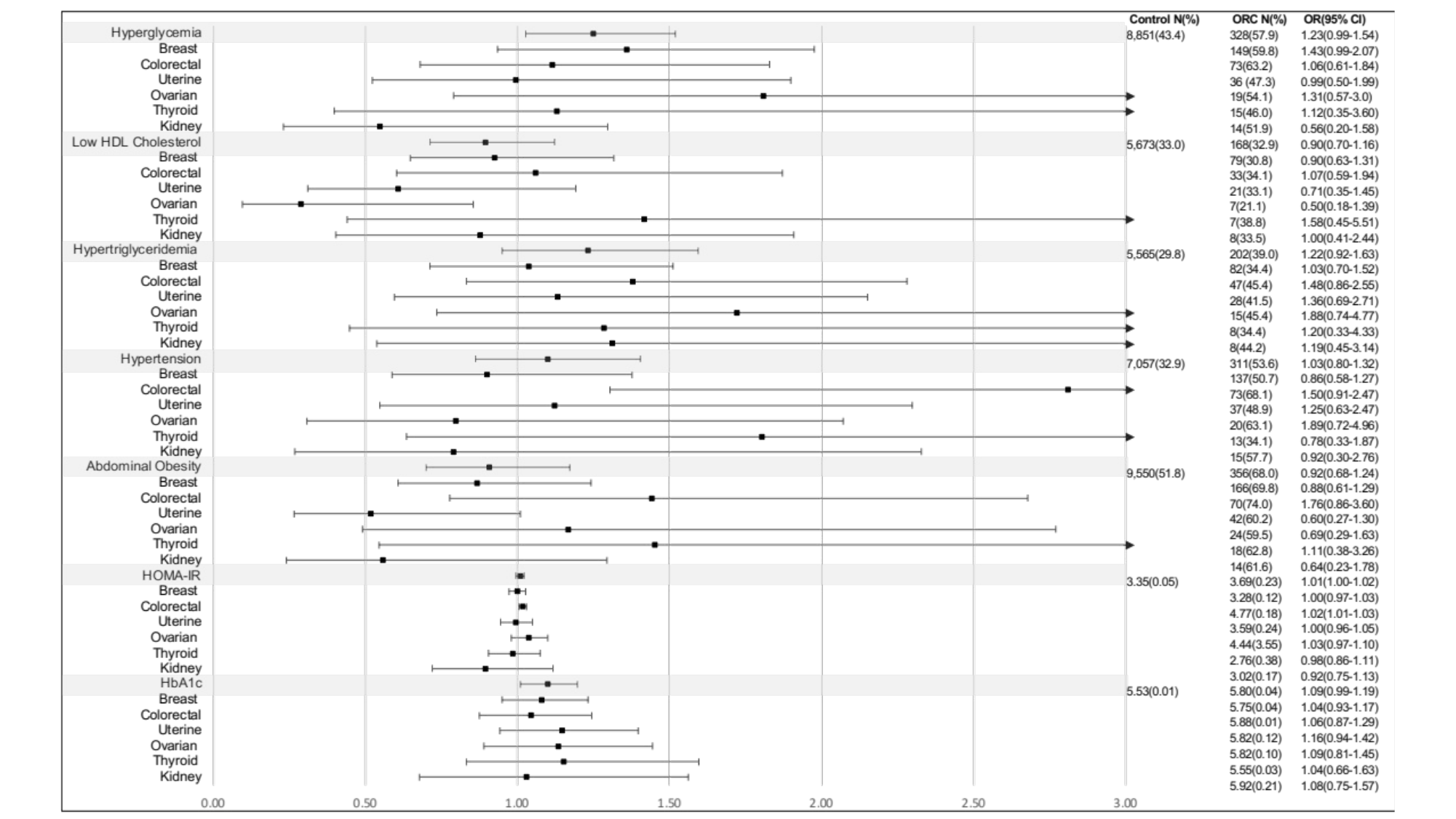

Supplement: Supplementary file 1 — Figure S1 [file CAM4-12-606-s002.pptx]
