# Supplementary material for: Metabolic dysfunction and obesity‐related cancer: Results from the cross‐sectional National Health and Nutrition Examination Survey
Source: Cancer Med. 2022 Jun 19;12(1):606–18. doi: 10.1002/cam4.4912 (PMC9844618; doi:10.1002/cam4.4912)
Supplement: Supplementary file 2 — Table S1‐S7 [file CAM4-12-606-s001.docx]

**SUPPLEMENTARY TABLES**

**Supplementary Table 1: Means, standard deviations, odd ratios (OR), and 95% confidence intervals (CI) of HOMA-IR and HbA1c in the National Health and Nutrition Examination Survey participants with a prior history of obesity-related cancer (ORC) diagnosis compared to cancer-free participants (N = 19,500).**

|  | **Cancer-free, n = 18,972** | | **ORC, n = 528** | | **OR***^a^* | **95% CI***^a^* |
| --- | --- | --- | --- | --- | --- | --- |
| **Metabolic Parameters** | *N/mean* | *%/SE* | *N/mean* | *%/SE* |  |  |
| **HOMA-IR: continuous** | 3.35 | 0.05 | 3.69 | 0.2 | 1.01 | 1.00-1.02 |
| **HOMA-IR: categorical** |  |  |  |  |  |  |
| Low risk (<2.5) | 9631 | 55.2 | 225 | 50.1 | REF | REF |
| Moderate risk (2.5-4.0) | 4063 | 21.2 | 121 | 23.3 | 1.19 | 0.88-1.61 |
| High risk (>4.0) | 4826 | 23.6 | 162 | 26.7 | 1.16 | 0.88-1.53 |
| **HbA1c: continuous** | 5.53 | 0.01 | 5.80 | 0.0 | 1.09 | 0.99-1.19 |
| **HbA1c: categorical** |  |  |  |  |  |  |
| Normal (<5.7) | 12942 | 74.5 | 246 | 52.3 | REF | REF |
| Pre-diabetes (5.7-6.4) | 4228 | 18.5 | 194 | 36.2 | 1.38 | 1.07-1.79 |
| Diabetes (>6.5) | 1754 | 6.8 | 86 | 11.1 | 1.30 | 0.91-1.85 |

*^a^Model adjusted for age, sex, race/ethnicity, education level, annual household income, smoking status, alcohol use, daily hours sedentary, weekly physical activity level, daily calorie intake, and survey year.*

*Abbreviations: SE: standard error, HOMA-IR: Homeostatic Model Assessment for Insulin Resistance.*

**Supplementary Table 2: Complete case analysis: adjusted odds ratios (OR) and 95% confidence intervals (CI) for obesity-related cancer (ORC) with metabolic syndrome (MetS) and metabolic syndrome score (MSS) in National Health and Nutrition Examination Survey participants (N = 16,870).**

|  | **Cancer-free** |  | **ORC** |  | **OR***^c^* | **95% CI***^c^* |
| --- | --- | --- | --- | --- | --- | --- |
|  | *N/mean* | *%/SE^c^* | *N/mean* | *%/SE^c^* |  |  |
| **MetS** |  |  |  |  |  |  |
| No | 10,408 | 65.4 | 193 | 50.4 | REF | REF |
| Yes | 6,022 | 34.6 | 247 | 49.6 | 1.01 | 0.77-1.32 |
| **MSS***^a^* | 1.91 | 0.0 | 2.54 | 0.1 | 1.05 | 0.96-1.16 |
| **MSS***^b^* |  |  |  |  |  |  |
| 0 | 2,934 | 19.9 | 23 | 7.1 | REF | REF |
| 1 or 2 | 7,474 | 45.5 | 170 | 43.2 | 1.6 | 0.93-2.75 |
| 3,4, or 5 | 6,022 | 34.6 | 247 | 49 | 1.51 | 0.86-2.64 |

*^a^MSS computed as a continuous variable, where each abnormal MetS parameter received a score of 1 and was summed to a total score out of 5.*

*^b^MSS computed as a categorial variable, where the total score out of 5 (number of abnormal MetS parameters for each participant) was compared to healthy participants with no abnormal MetS parameters (ref = 0).*

*^c^Model adjusted for age, sex, race/ethnicity, education level, annual household income, smoking status, alcohol use, daily hours sedentary, weekly physical activity level, daily calorie intake, and survey year.*

*Abbreviations: SE: standard error.*

**Supplementary Table 3: Adjusted odds ratios (OR) and 95% confidence intervals (CI) for obesity-related cancer (ORC) with combinations of two abnormal metabolic syndrome parameters in National Health and Nutrition Examination Survey participants with a metabolic syndrome score of two***^a^* **(N = 4,445)** *^a^***.**

| **Combination** | **OR***^b^* | **95% CI***^b^* |
| --- | --- | --- |
| Hyperglycemia, Low-HDL | 0.29 | 0.03-2.69 |
| Hyperglycemia, Hypertriglyceridemia | 1.70 | 0.28-10.29 |
| Hyperglycemia, Hypertension | 1.15 | 0.46-2.86 |
| Hyperglycemia, Central Obesity | 1.19 | 0.44-3.16 |
| Low-HDL, Hypertriglyceridemia | 1.70 | 0.40-7.17 |
| Low-HDL, Hypertension | 1.53 | 0.33-7.17 |
| Low-HDL, Central Obesity | 0.15 | 0.03-0.87 |
| Hypertriglyceridemia, Hypertension | 2.46 | 0.60-10.16 |
| Hypertriglyceridemia, Central Obesity | 1.24 | 0.40-3.88 |
| Hypertension, Central Obesity | 1.26 | 0.55-2.90 |

*^a^Metabolic syndrome score (MSS) of 2 = two abnormal metabolic parameters.*

*^b^Model adjusted for age, sex, race/ethnicity, education level, annual household income, smoking status, alcohol use, daily hours sedentary, weekly physical activity level, daily calorie intake, and survey year.*

**Supplementary Table 4: Demographic information for National Health and Nutrition Examination Survey participants with a prior history of obesity-related cancer (ORC) and normal weight and overweight participants without a prior history of cancer (N = 19,500).**

|  | **No cancer,**  **n = 6,079** |  | **No cancer,**  **n = 12,621** |  | **ORC cases, n = 528** |  |
| --- | --- | --- | --- | --- | --- | --- |
|  | ***Normal Weight***  *N/mean %/SE* | | ***Overweight***  *N/mean %/SE* | | *N/mean %/SE* | |
| **Age in years*^a^*** |  |  |  |  |  |  |
| <50 | 3,937 | 69.2 | 6,723 | 59.1 | 72 | 19.3 |
| 50-59 | 708 | 14.3 | 2,047 | 18.8 | 70 | 14.4 |
| 60-69 | 667 | 8.6 | 2,120 | 13.0 | 136 | 25.3 |
| 70-79 | 422 | 4.7 | 1,178 | 6.4 | 143 | 24.5 |
| ≥80 | 345 | 3.3 | 553 | 2.6 | 107 | 16.5 |
| **Sex** |  |  |  |  |  |  |
| Female | 3,077 | 54.4 | 6,196 | 47.7 | 458 | 90.4 |
| Male | 3,002 | 45.6 | 6,425 | 52.3 | 70 | 9.6 |
| **Race/ethnicity** |  |  |  |  |  |  |
| White (non-Hispanic) | 2,696 | 68.5 | 5,186 | 66.3 | 313 | 78.9 |
| Black (non-Hispanic) | 1,109 | 9.7 | 2,783 | 12.9 | 83 | 8.7 |
| Mexican American/ Hispanic | 1,381 | 11.0 | 3,959 | 15.9 | 108 | 8.5 |
| Other*^b^* | 893 | 10.7 | 693 | 4.9 | 24 | 3.8 |
| **Income** |  |  |  |  |  |  |
| <$35,000 | 2,872 | 38.0 | 5,904 | 36.5 | 267 | 43.0 |
| $35,000-$75,000 | 1,628 | 29.8 | 3,814 | 33.4 | 153 | 32.5 |
| >$75,000 | 865 | 18.3 | 1,598 | 18.0 | 59 | 13.1 |
| **Education** |  |  |  |  |  |  |
| <High School | 1,678 | 18.2 | 5,851 | 55.5 | 155 | 20.8 |
| High School | 1,416 | 22.6 | 3,010 | 24.8 | 125 | 27.7 |
| >High School | 2,975 | 59.1 | 3,751 | 19.6 | 248 | 51.6 |
| **Smoking** |  |  |  |  |  |  |
| Never | 2,948 | 50.9 | 6,624 | 52.9 | 284 | 51.6 |
| Former | 1,024 | 18.1 | 3,127 | 25.7 | 180 | 34.7 |
| Current | 1,389 | 25.6 | 2,330 | 19.5 | 63 | 13.6 |
| **Total daily calorie intake** | 2,097 | 16.7 | 2,087 | 11.4 | 1649 | 30 |
| **Physical activity level** |  |  |  |  |  |  |
| No/low activity | 1,909 | 26.3 | 4,526 | 30.7 | 243 | 38.8 |
| Moderate/vigorous activity | 4,108 | 72.8 | 7,906 | 68.1 | 263 | 57.6 |
| **Sedentary Hours** |  |  |  |  |  |  |
| < 5 hours | 3,633 | 60.7 | 7,156 | 54.7 | 284 | 53.6 |
| ≥ 5 hours | 2,443 | 39.2 | 5,462 | 45.3 | 244 | 46.4 |
| **Alcohol Use** |  |  |  |  |  |  |
| No | 1,261 | 18.1 | 2,993 | 20.5 | 191 | 33.4 |
| Yes | 3,193 | 60.8 | 7,194 | 62.2 | 254 | 50.6 |
| **Menopausal status***^c^* |  |  |  |  |  |  |
| Premenopausal | 1,604 | 56.0 | 2,467 | 42.8 | 33 | 8.3 |
| Postmenopausal | 1,218 | 36.7 | 3,216 | 49.9 | 397 | 87.0 |
| **HRT use***^c^* |  |  |  |  |  |  |
| No | 1,958 | 69.5 | 4,161 | 68.1 | 270 | 54.9 |
| Yes | 493 | 17.6 | 1,228 | 22.2 | 157 | 39.7 |
| **Metabolic Parameters** |  |  |  |  |  |  |
| Hyperglycemia | 1,799 | 25.6 | 6,906 | 52.1 | 328 | 57.9 |
| Low-HDL | 1,042 | 18.8 | 4,541 | 40.0 | 168 | 32.1 |
| Hypertriglyceridemia | 986 | 15.7 | 4,487 | 36.7 | 202 | 40 |
| Hypertension | 1,577 | 21.8 | 5,342 | 38.2 | 311 | 53.6 |
| Abdominal Obesity | 429 | 7.8 | 9,079 | 73.7 | 356 | 68 |
| HOMA-IR | 1.67 | 0.02 | 4.16 | 0.06 | 3.69 | 0.23 |
| HbA1C (%) | 5.32 | 0.01 | 5.64 | 0.01 | 5.8 | 0.04 |

*^a^Participants with age ≥ 85 are recorded as age = 85 in the NHANES datasets.*

*^b^The “other” race category in NHANES includes non-Hispanic Asian and all non-Hispanic persons that reported races other than Black, Asian, or White.*

*^c^Among* *female study participants only.*

**Supplementary Table 5:** **Odd ratios (OR), and 95% confidence intervals (CI) of metabolic syndrome (MetS) and metabolic syndrome score (MSS) in National Health and Nutrition Examination Survey female participants with a prior history of obesity-related cancer (ORC) diagnosis compared to cancer-free participants (N = 9,876)** *^c^***.**

|  | **Cancer-free** |  | **ORC** |  | **OR***^d^* | **95% CI***^d^* |
| --- | --- | --- | --- | --- | --- | --- |
|  | *N/mean* | *%/SE^c^* | *N/mean* | *%/SE^c^* |  |  |
| **MetS** |  |  |  |  |  |  |
| No | 6,044 | 67.4 | 217 | 54.6 | REF | REF |
| Yes | 3,374 | 32.6 | 241 | 45.4 | 0.89 | 0.68-1.16 |
| **MSS***^a^* | 1.84 | 0.0 | 2.43 | 0.1 | 1.03 | 0.93-1.13 |
| **MSS***^b^* |  |  |  |  |  |  |
| 0 | 1,759 | 21.9 | 24 | 7.1 | REF | REF |
| 1 or 2 | 4,285 | 45.5 | 193 | 47.5 | 1.73 | 1.04-2.89 |
| 3,4, or 5 | 3,374 | 32.6 | 241 | 45.4 | 1.44 | 0.85-2.43 |

*^a^MSS computed as a continuous variable, where each abnormal MetS parameter received a score of 1 and was summed to a total score out of 5.*

*^b^MSS computed as a categorial variable, where the total score out of 5 (number of abnormal MetS parameters for each participant) was compared to healthy participants with no abnormal MetS parameters (ref = 0).*

*^c^Model adjusted for age, sex, race/ethnicity, education level, annual household income, smoking status, alcohol use, daily hours sedentary, weekly physical activity level, daily calorie intake, survey year, menopausal status, and hormone replacement therapy.*

*Abbreviations: SE: standard error.*

**Supplementary Table 6:** **Odd ratios (OR), and 95% confidence intervals (CI) of metabolic syndrome (MetS) and metabolic syndrome score (MSS) in National Health and Nutrition Examination Survey female participants with a history of breast cancer and post-menopausal breast cancer diagnoses, compared to cancer-free participants (N = 9,876).**

|  | **OR***^a^* | **95% CI***^a^* |
| --- | --- | --- |
| **MetS** |  |  |
| Breast*^b^* | 0.80 | 0.55-1.16 |
| Breast*^c^* | 0.80 | 0.52-1.21 |
| **MSS** |  |  |
| Breast*^b^* | 0.98 | 0.86-1.11 |
| Breast*^c^* | 0.96 | 0.82-1.13 |

*^a^Model adjusted for age, sex, race/ethnicity, education level, annual household income, smoking status, alcohol use, daily hours sedentary, weekly physical activity level, daily calorie intake, survey year, menopausal status, and hormone replacement therapy.*

*^b^All breast cancer cases.*

*^c^Post-menopausal breast cancer cases only.*
